# T51. TREATMENT OF NEGATIVE SYMPTOMS OF SCHIZOPHRENIA WITH TRANSCRANIAL CURRENT STIMULATION (TDCS): RESULTS OF RANDOMIZED, DOUBLE-BLINDED, SHAM-CONTROLLED TRIAL

**DOI:** 10.1093/schbul/sby016.327

**Published:** 2018-04-01

**Authors:** Leandro Valiengo, Pedro Gordon, Mauricio Serpa, Acioly Lacerda, Wagner Gattaz, Martinus Van de Bilt, Helio Helkis, Andre Brunoni

**Affiliations:** 1 University of Sao Paulo; 2 Centro de Pesquisa e Ensaios Clínicos Sinapse-Bairral

## Abstract

**Background:**

The negative symptoms of schizophrenia cause significant distress and impairment. The treatment of them is a challenge, with medications having none or little effect. So, new treatments are necessary for this condition. The aim of the study was to ascertain the efficacy of tDCS in treating negative symptoms of schizophrenia

**Methods:**

This study was designed to be a randomized, sham-controlled, double-blinded trial using tDCS for the treatment of negative symptoms of schizophrenia. One-hundred (here we analyzed only 70% of the sample, the remaining will be presented at the meeting) patients will be enrolled and submitted to ten tDCS session over the left dorsolateral prefrontal cortex (anodal stimulation) and left temporo-parietal junction-left (cathodal stimulation), over 5 consecutive days, with 2 mA of current. Participants were assessed with clinical and neuropsychological tests before and after the intervention. The primary outcome was change (over time and across groups) in the scores of the Negative Subscale of Positive and Negative Symptoms Syndrome (PANSS). Our secondary outcomes consist of others scales as SANSS (Scale of Assessment of Negative Symptoms), Calgary and the AHRS (Auditory Hallucinations Rating Scale).

**Results:**

From 70% of the sample the active tDCS was significantly superior to sham at endpoint at 6 weeks by negative sub scale of PANSS (mean difference, 3,5 points; SD=6.2; P<.05). The total PANSS and the hallucinations scale had no differences between both groups. The other times of analysis were not found differences between sham and active groups. The others scales (Calgary and SANSS have not being evaluated yet).

**Discussion:**

The results of our studies suggests a potential role of tDCS for the treatment of negative symptoms of schizophrenia. The effect size was small. This is the biggest study with tDCS for treating negative symptoms of schizophrenia until now. At the meeting all the data will be analyzed (100 patients), it these could change our preliminary results.

